# A robust and efficient algorithm for the shape description of protein structures and its application in predicting ligand binding sites

**DOI:** 10.1186/1471-2105-8-S4-S9

**Published:** 2007-05-22

**Authors:** Lei Xie, Philip E Bourne

**Affiliations:** 1 San Diego Supercomputer Center, University of California, San Diego, 9500 Gilman Drive, La Jolla, CA 92093, USA; 2 Department of Pharmacology, University of California, San Diego, 9500 Gilman Drive, La Jolla, CA 92093, USA

## Abstract

**Background:**

An accurate description of protein shape derived from protein structure is necessary to establish an understanding of protein-ligand interactions, which in turn will lead to improved methods for protein-ligand docking and binding site analysis. Most current shape descriptors characterize only the local properties of protein structure using an all-atom representation and are slow to compute. We need new shape descriptors that have the ability to capture both local and global structural information, are robust for application to models and low quality structures and are computationally efficient to permit high throughput analysis of protein structures.

**Results:**

We introduce a new shape description that requires only the C*α *atoms to represent the protein structure, thus making it both fast and suitable for use on models and low quality structures. The notion of a *geometric potential *is introduced to quantitatively describe the shape of the structure. This geometric potential is dependent on both the global shape of the protein structure as well as the surrounding environment of each residue. When applying the geometric potential for binding site prediction, approximately 85% of known binding sites can be accurately identified with above 50% residue coverage and 80% specificity. Moreover, the algorithm is fast enough for proteome-scale applications. Proteins with fewer than 500 amino acids can be scanned in less than two seconds.

**Conclusion:**

The reduced representation of the protein structure combined with the geometric potential provides a fast, quantitative description of protein-ligand binding sites with potential for use in large-scale predictions, comparisons and analysis.

## Background

The 3D structure of a protein is an essential component in elucidating biological functions at the molecular level. Protein-ligand binding sites and their interactions with binding partners provide strong correlations between structure and function and are thus critical for addressing a wide range of fundamental and practical problems in biology. Knowledge of protein-ligand binding sites provides not only critical clues in elucidating the relationships to evolution, structure and function, but also contributes to drug discovery. Knowledge of such sites may be used to identify and validate drug targets, prioritize and optimize drug leads, rationalize small molecule screening and docking, guide medicinal chemistry efforts and computationally evaluate ADME/Tox properties of preclinical drugs. To derive knowledge of the ligand binding site from the exponentially increasing amount of structural data, it is critical to develop a sensitive and robust algorithm that can identify and characterize the ligand binding sites of proteins on a proteome-wide scale.

Shape descriptors representing protein structure, such as depth [1,2], surface curvature [3], extreme elevation [4], solid angle [5], surface area [6] and volume [6], have been used extensively to identify, study and compare protein-ligand interactions, protein-protein interactions and the respective binding sites. For example, the extreme elevation approach is used for geometric alignment during protein docking [7]. The match of small molecules to protein binding sites has been studied using the molecular shape complementarities of solid angles [5,8]. Besides predictions of ligand orientations, one of the biggest challenges in any docking study is to obtain an accurate estimate of the binding affinity while including the intrinsic flexibility of the protein and the ligand. Soft docking provides a solution to these problems [9]. The adaptive scoring function for soft docking requires a defined "hard" and "soft" interaction range between the protein and the ligand. Furthermore, the accuracy in estimating binding affinity can be dramatically improved with the docking score index (DSI) from multiple ligand, multiple protein docking [10]. The use of a virtual ligand has been proposed to extend the DSI schema for genome-wide high throughput screening [11]. The success of the proposed DSI method critically depends on the generation of the virtual ligand, which is a negative image of the ligand binding site. It is still an open question how to define such a virtual ligand, or equivalently the boundary to the ligand binding site. Geometry based methods are very useful in detecting pockets and cavities within the protein structure [6,12-17], and can be applied independently or combined with other evolutionary [18-21] or physical based methods [22,23]. Although these existing methods [2,6,12-17] can locate the binding pockets accurately, the accurate definition of the pocket boundary remains rather poor [24]. This inaccurate description limits further application for protein-ligand docking and functional site comparison. Moreover, the geometrical measurement of pockets and cavities using shape descriptors such as volume and curvature alone is not a good indicator to distinguish true binding pockets from false positives [2].

Nevertheless, geometric constraints have been used extensively to assess the similarity between functional sites [25]. Most of these studies focus on the local shape of the protein using distance, curvature, and side chain orientation that are sensitive to conformational changes of either the side chains or backbone. To extend the scope of functional site comparison algorithms, it is necessary to develop topological and geometric invariants that are less sensitive to the flexibility and uncertainty inherent in the protein structure, yet still provide a useful metric. In summary, there are several drawbacks in using conventional shape descriptors for the protein structure when applied to ligand binding studies. First, most of these measurements capture the local property of the protein structure and do not distinguish between the ligand binding and non-binding site. Second, some shape descriptors require an all-atom representation of the protein structure, making the algorithm computationally intensive. Finally, existing algorithms are sensitive to conformational changes in the protein structure and are intolerant to the uncertainty inherent in homology models and low resolution structures. Given these shortcomings we propose a new method for protein shape description of the protein structure that is scalable to a large data set of proteins yet robust enough to handle the intrinsic properties of protein flexibility. Inherent in the method is the provision of the location and boundary of any binding pocket, thus providing a new approach to the study of protein-ligand interactions.

Details are given in the Methods section, but in summary the topological relationships among C*α *atoms in the protein are established using Delaunay tessellation [26] of these atoms in 3D space. From the Delaunay tessellation of the reduced representation of the protein structure, shape descriptors such as the direction of the C*α *atom relative to the surface can be determined. The notion of a *geometric potential *is further introduced to quantitatively characterize the so-called shape formed by the set of C*α *atoms. The geometric potential is analogous to the hydrophobicity or electrostatics potential in that it is dependent on both the global shape of the protein structure as well as the surrounding environment of the residue. The geometric potential has been successfully applied in a new algorithm for ligand binding site comparison [L. Xie and P.E. Bourne, "Detecting evolutionary linkages across fold and functional space with sequence order independent profile-profile alignments," submitted]. Here we focus on a detailed description of the geometric potential algorithm and applications that predict the ligand binding site.

## Results

### Features of the geometric potential

As described in the Methods section a new shape descriptor, the geometric potential, has been developed to characterize protein structures. The geometric potential is used to distinguish the ligand binding site from non-ligand binding sites. Figure 4 shows the distribution of the geometric potential for residues (Figure 4A) and residue clusters (Figure 4B) that are involved and not involved in the ligand binding from the benchmark dataset, respectively. The geometric potential for the majority of non-binding site residues and residue clusters is close to zero, whereas it is centered around 50 for binding site residue clusters, and clearly separated from non-binding site residue clusters. This discrimination implies that the geometric potential can be used as a feature to distinguish ligand binding and non-binding sites.

Unlike local measurements of geometric properties such as solvent accessible surface area, the standard deviation for the geometric potential for the majority of binding sites is quite small (around 5–10 on a 100 scale), as shown in Figure 5. The standard deviation in relative solvent accessible surface area with a probe radius of 1.4 Å is above 15.0 on a 100 scale for most of the binding sites. Thus, the geometric potential proves to be synonymous with the binding site and can be used to segment proteins into biological meaningful residue clusters with clearly defined boundaries.

Figure 6 are examples showing the reduced protein structure representations of C*α *atoms and their associated protein boundaries. The left and right images are holo and apo proteins, respectively. Each example exhibits large conformational changes in the binding sites upon ligand binding [27]. At the same time, the RMSD is greater than 2.0 Å between the holo and apo structure across the whole protein. The values of the computed geometric potentials for each residue are color mapped to the structure. The highest and lowest value is colored red and blue, respectively. As shown in Figure 6, the overall patterns of the geometric potential in the holo and apo protein are similar, indicating that the algorithm is not sensitive to the conformation changes found in each structure. The geometric potential for the residues involved in binding are relatively high in most cases with immunoglobulin 48g7 germline fab being an exception. In this case the known binding site is located in the interface of a homo-dimer and is quite shallow. It is generally not easy to identify shallow binding sites using purely geometric based methods. It is noted that there are few examples where large conformational changes takes place across the whole protein upon ligand binding [28]. Among 20 proteins whose binding sites show large conformational changes [27], only 4 cases have an RMSD larger than 2.0 Å between holo and apo proteins. All of these cases are included in Figure 6.

### Rank accuracy

The predicted ligand binding sites were ranked from highest to lowest based on the geometric potential for each protein (Figure 7). Whether or not a binding site is predicted correctly depends on the tolerance of percentage of matched residues between predicted and known binding site. For predicted binding site residues with above 50% residues in common with the known binding site (i.e., sensitivity above 50%), approximately 55.0% and 75% of known ligand binding sites are ranked as the top one and top three, respectively. Alternatively, if the goal is to locate the ligand binding site, above 70.0% and 90.0% of test cases can be correctly predicted as one of the top one and three, respectively with a sensitivity above 10%. These results further indicate that the geometric potential is a useful feature to assess the significance of predicted binding sites.

### Boundary sensitivity and specificity

As stated previously, one weakness of most existing algorithms is the inability to accurately define the boundary of the predicted binding site. Both sensitivity and specificity are important to define the boundary of the prediction. It is relatively easy to achieve high sensitivity and cover all of the binding site residues. In an extreme example, all residues in a protein can be predicted as a binding site so that the sensitivity is 100%. However, it is not trivial to predict the residue coverage with both high sensitivity and specificity. As shown in Figure 8, for the benchmark dataset the specificity of our algorithm is high, with greater than 90% of known ligand binding sites predicted with residue specificity above 80% (Figure 8A). At the same time over 80% of known ligand binding sites are correctly predicted with sensitivity above 50%. Taking both sensitivity and specificity into account, as shown in Figure 9, around 85% of binding sites can be identified with sensitivity above 50% and specificity above 80%. Thus, besides accurately identifying the location of the ligand binding site, the algorithm also clearly defines the boundary. A clear boundary definition is important for application to protein-ligand docking, virtual screening, and ligand binding site comparison.

### Comparison to other algorithms

The convex hull and related *α*-shape algorithms (see Methods) have been applied by others to identify ligand binding pockets [6,15]. These methods were developed on the basis of the all-atom representation of the protein structure. Although it is possible to apply them to the C*α *only representation, extensive work on parameterization may be required. For example, it is not trivial to define the optimum value of one parameter, the probe radius, for the C*α *only representation using CASTp [29]. The standard setting of 1.4 Å is too small to define the surface of the protein. As a result, extended tunnels are formed across the protein. Conversely, a probe with a larger radius will leave a number of binding site residues inaccessible. Figure 10 shows an example of ligand binding prediction by CASTp [29]. The prediction is quite sensitive to the choice of the probe radius using a C*α *only representation. The predicted binding site covers half of the protein with a probe radius of 1.4 Å because of the formation of tunnels. The sensitivity and specificity of the correctly predicted binding pocket are close to the all-atom representation when the probe radius is 2.8 Å (volume 4825.3 Å^3^, area 2139 Å^2^). However, another pocket with a similar volume (4181.4 Å^3^) and area (2573 Å^2^) to the correct one is identified. It is not a real pocket but rather forms an interface between helices. Increasing the probe radius to 5.6 Å results in a number of the binding site residues being buried, thus the sensitivity of the predicted binding site drops significantly. Moreover, the detected pockets from these algorithms could only be ranked with their volumes and areas. These shape descriptors are less significant in distinguishing the binding sites [2]. As a comparison, the ligand binding site from the same protein is ranked by the geometric potential at the top one with sensitivity of 100% and specificity of 90%.

### Time complexity

The algorithm is implemented using the JAVA programming language and tested on a non-dedicated personal computer with 3.0 GHz single processor and 2.0 GB RAM. As shown in Figure 11, the time complexity is approximated to O(*n*^2^), but fast enough for real applications. For most proteins consisting of fewer than 500 amino acids, the whole structure can be scanned in less than 2.0 seconds.

## Discussion

### General concept of the geometric potential

The current implementation of the geometric potential is computationally straightforward and conceptually analogous to a residue's electrostatic potential or hydrophobicity, both of which depend on both the global shape of the protein structure and the surrounding environment of the residue. Suppose that the protein is an insulator with a homogenous hydrophilic surface and surrounded by solvent with positive charge. Each of the residues represented by C*α *atoms carries a unit of negative charge. The electrostatic potential essentially reflects the residue's geometrical characteristics at the surface and in the pocket/cavity. The electrostatic potential is most negative in a closed cavity, less negative in an open pocket, almost neutral on a flat surface, and most positive on a convex surface. Thus, the geometric potential can, in theory, be computed using rigorous energy-based methods such as Poisson-Boltzmann for the electrostatic free energy [30] on a protein structure with a reduced representation of residues and charges. In other words, if non-specific interactions such as van der Waals interactions are used to predict ligand binding sites [23,31], they provide almost identical information to that derived from geometric properties. However, the direct energy based method usually requires an all-atom representation of the protein and the accurate estimation of the interaction energy is not trivial. Consequently, it is possible to integrate the geometric and topological properties characterized by the geometric potential with energy-based physical potentials into a unified framework to study protein-ligand binding.

### Relationship to other algorithms

The convex hull and related *α*-shape algorithm (see methods) have been applied by others to identify ligand binding pockets [6,15]. Other approaches usually require an all-atom representation of the protein structure. Here, with one simple parameter which uses the radius of the circumscribed sphere from the Delaunay tessellation, a C*α *atom representation is sufficient. As a result, the algorithm is theoretically two orders of magnitude faster than all atom approaches because the time complexity of 3D Delaunay tessellation is *O*(*n*^2^) where *n *is the number of input points. Moreover, as shown, the algorithm is not sensitive to conformational changes of the protein, especially the side chains.

The geometric potential provides a more robust quantitative measurement of the geometrical and topological properties of the pocket and cavity taking into account both the global and local environment surrounding the amino acid residue and hence offers advantages over more conventional measurements such as depth [1,2], travel depth [1], surface curvature [3], surface area [6] and volume [6]. Recent studies have shown that depth is a more important attribute than curvature, volume and other shape descriptors in distinguishing drugable binding sites [2]. However, the depth or the travel depth of an amino acid residue in a pocket cannot indicate whether the residue resides in a narrow or wide open pocket with the same depth. Moreover, the depth cannot distinguish flat and convex surfaces if they have the same depths. The geometric potential distinguishes these cases. The depth used in our studies to initialize the geometric potential is quite simple and not as accurate as the travel depth proposed by Coleman et al. [1]. However, it is straightforward to implement and incorporate the travel depth concept into our approach by replacing the distance to the closed plane with the travel depth during computing the geometric potential. Other geometric or topological measurements such as the distance to the centric of the protein [13] and closeness centrality [32], which are able to distinguish ligand binding and non-binding sites, can also be used to initialize the geometric potential.

### Limitations of the algorithm and future work

Ligand binding is primarily a physics phenomenon, depending on fundamental thermodynamics and kinetics. Therefore, the ligand binding site may be best studied with an energy-based method, such as protein-ligand docking [33,34], grid potential mapping [31], or solvent/fragment mapping [35-37]. However, docking is not only time consuming computationally, but also inaccurate in estimating the binding energy. Geometric properties of the protein structure, such as pockets and cavities, provide rational constraints to address the docking problem. Another important constraint on the ligand binding site comes from evolution. The identification of conserved residues from sequence analysis significantly reduces the search space thus aiding the location of the ligand binding site. Therefore, the identification of the location and boundary of the ligand binding site is best achieved by integrating protein features associated with geometry, evolution and energy. While not yet completed, the concept of the geometric potential as described here provides a quantitative framework to combine these sources of information.

The approach presented here is extensible and can be used to predict protein-protein binding sites. The majority of these sites are formed from relatively flat surfaces and thus their geometric potentials are less distinguishable than those of pockets and cavities. Solvent vectors have previously been used to define protein-protein interaction interfaces [38]. The global direction of the residue from our algorithm provides a more robust method than the solvent vector to cluster the residues involved in protein-protein ligand binding and further define its boundary. Moreover, if the real hydrophobicity and/or the electrostatic energy are used as the initial value for the geometric potential, it is possible to quantitatively distinguish the protein-protein binding site.

## Conclusion

We introduce a new efficient and robust algorithm that quantitatively characterizes the geometric properties of the protein structure. The geometric potential is dependent on both the global shape of the protein structure as well as the surrounding environment of the residue. In this sense it is analogous to the hydrophobicity or electrostatic potential. When applying the geometric potential to ligand binding site prediction, the top three predictions contain more than 75% of the known binding sites and provide at least 50% coverage of the ligand binding site residues. Approximately 85% of known ligand binding sites can be accurately identified with above 50% residue coverage and 80% specificity for all predicted binding sites. Moreover, the algorithm is fast enough for proteome-scale applications. Proteins with fewer than 500 amino acids can be scanned in less than two seconds. The algorithm provides a framework for integrating evolution, energy, and further geometry-based parameters to study protein-ligand interactions on a proteome-wide scale.

## Methods

### Benchmarks

A data set of protein-ligand binding sites is built from protein chains in the Protein Data Bank (PDB) [39] with known 3D protein structures with bound ligands. Only small organic molecules are considered as ligands; DNA, RNA, peptides and metals are excluded. The non-redundant set of protein chains are selected with sequence identities less than 90%. Only x-ray structures are included in our test data set. The final benchmark dataset contains a total of 5263 enzyme and non-enzyme polypeptide chains, as defined as best as possible by the presence or absence of EC numbers.

For each protein-ligand complex, the residues involved in ligand binding are those where any of the atoms in a residue are within a 10.0 Å radius of any ligand atom and the line segment connecting these two atoms does not intersect with other protein atoms. There are 7,570 binding sites found in the 5263 chains involving 48,819 binding residues and 1,414,293 non-binding residues, respectively. In addition, 54,826 residue clusters are randomly generated from non-binding site residues as negative controls. To generate the clusters, one of the solvent accessible residues that are not involved in the ligand binding on the protein is randomly selected as the center. Then it, with all of its neighboring solvent accessible residues within a 10.0 Å radius, defines the cluster. Clusters are selected so as not to overlap.

### Overview of the algorithm

**Figure 1:**
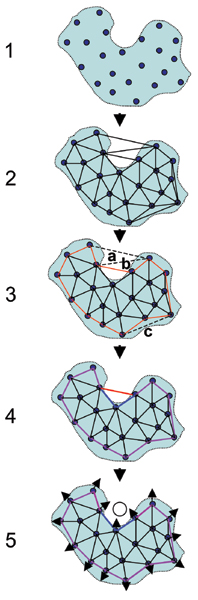
**Overview of the algorithm**. The solid body and circles indicate an all-atom and Cá atom representation, respectively. Open circles are virtual atoms determined by the algorithm. (1) Step 1: the protein structure is represented as Cá atoms. (2) Step 2: Cá atoms are Delaunay tessellated. The convex hull is determined at the same time. (3) Step 3: the environmental boundary (red solid lines) is determined from the Delaunay tessellation by peeling off the tetrahedra (triangles labeled as a, b, and c) with edge lengths larger than 30.0 Å (black dashed lines) starting from the convex hull. (4) Step 4: the protein boundary (blue and purple solid lines). The purple lines are overlapped with the environmental boundary and determined from the Delaunay tessellation by removing tetrahedra with circumscribed sphere radius larger than 7.5 Å. (5) Step 5–7: shape descriptors such as residue surface direction and geometric potential for each Cá atom position are computed and ligand binding sites and virtual atoms (open circle) are predicted.

The algorithm consists of the following steps (Figure 1).

#### Step 1. Representation of the protein structure

The protein structure is represented by C*α *atoms only, making it computationally efficient and applicable to low resolution structures and homology models on a proteome-wide scale.

#### Step 2. Delaunay tessellation of the C*α *atoms

The structure is Delaunay tessellated using a convex hull algorithm [40] implemented in the Qhull package. As a result, the structure is partitioned into a set of tetrahedra. A unique circumscribed sphere is defined for each tetrahedron such that its four vertices touch the surface of the sphere. In doing so the following determinant is obeyed:

|x2+y2+z2xyz1x12+y12+z12x1y1z11x22+y22+z22x2y2z21x32+y32+z32x3y3z31x42+y42+z42x4y4z41|=0     (1)
 MathType@MTEF@5@5@+=feaafiart1ev1aaatCvAUfKttLearuWrP9MDH5MBPbIqV92AaeXatLxBI9gBaebbnrfifHhDYfgasaacH8akY=wiFfYdH8Gipec8Eeeu0xXdbba9frFj0=OqFfea0dXdd9vqai=hGuQ8kuc9pgc9s8qqaq=dirpe0xb9q8qiLsFr0=vr0=vr0dc8meaabaqaciaacaGaaeqabaqabeGadaaakeaadaabdaqaauaabeaafuaaaaaabaGaemiEaG3aaWbaaSqabeaacqaIYaGmaaGccqGHRaWkcqWG5bqEdaahaaWcbeqaaiabikdaYaaakiabgUcaRiabdQha6naaCaaaleqabaGaeGOmaidaaaGcbaGaemiEaGhabaGaemyEaKhabaGaemOEaOhabaGaeGymaedabaGaemiEaG3aa0baaSqaaiabigdaXaqaaiabikdaYaaakiabgUcaRiabdMha5naaDaaaleaacqaIXaqmaeaacqaIYaGmaaGccqGHRaWkcqWG6bGEdaqhaaWcbaGaeGymaedabaGaeGOmaidaaaGcbaGaemiEaG3aaSbaaSqaaiabigdaXaqabaaakeaacqWG5bqEdaWgaaWcbaGaeGymaedabeaaaOqaaiabdQha6naaBaaaleaacqaIXaqmaeqaaaGcbaGaeGymaedabaGaemiEaG3aa0baaSqaaiabikdaYaqaaiabikdaYaaakiabgUcaRiabdMha5naaDaaaleaacqaIYaGmaeaacqaIYaGmaaGccqGHRaWkcqWG6bGEdaqhaaWcbaGaeGOmaidabaGaeGOmaidaaaGcbaGaemiEaG3aaSbaaSqaaiabikdaYaqabaaakeaacqWG5bqEdaWgaaWcbaGaeGOmaidabeaaaOqaaiabdQha6naaBaaaleaacqaIYaGmaeqaaaGcbaGaeGymaedabaGaemiEaG3aa0baaSqaaiabiodaZaqaaiabikdaYaaakiabgUcaRiabdMha5naaDaaaleaacqaIZaWmaeaacqaIYaGmaaGccqGHRaWkcqWG6bGEdaqhaaWcbaGaeG4mamdabaGaeGOmaidaaaGcbaGaemiEaG3aaSbaaSqaaiabiodaZaqabaaakeaacqWG5bqEdaWgaaWcbaGaeG4mamdabeaaaOqaaiabdQha6naaBaaaleaacqaIZaWmaeqaaaGcbaGaeGymaedabaGaemiEaG3aa0baaSqaaiabisda0aqaaiabikdaYaaakiabgUcaRiabdMha5naaDaaaleaacqaI0aanaeaacqaIYaGmaaGccqGHRaWkcqWG6bGEdaqhaaWcbaGaeGinaqdabaGaeGOmaidaaaGcbaGaemiEaG3aaSbaaSqaaiabisda0aqabaaakeaacqWG5bqEdaWgaaWcbaGaeGinaqdabeaaaOqaaiabdQha6naaBaaaleaacqaI0aanaeqaaaGcbaGaeGymaedaaaGaay5bSlaawIa7aiabg2da9iabicdaWiaaxMaacaWLjaWaaeWaaeaacqaIXaqmaiaawIcacaGLPaaaaaa@9A4E@

Where (*x*_*i*_, *y*_*i*_, *z*_*i*_) with *i *= 1, 2, 3, and 4 are the coordinates of the points of the tetrahedron from the Delaunay tessellation.

The effect of Delaunay tessellation is to generate a convex hull surrounding the C*α *atoms of the protein.

#### Step 3. Determination of the environmental boundary of the protein structure

The environmental boundary is defined as an outside layer that contains all of the C*α *atoms of the protein (red solid lines in Figure 1). It is computed by iteratively peeling off the tetrahedra that include edges longer than 30.0 Å layer by layer starting from the convex hull. The value of 30.0 Å is an empirical estimate for the maximum size of a ligand binding pocket. As a result, some of the triangles on the original convex hull are removed and the C*α *atoms of the protein are surrounded with the newly formed triangles resulting from the removal of tetrahedra with edge length longer than 30.0 Å and the remaining triangles on the convex hull. This set of triangles forms the environmental boundary which contains both the protein and any potential ligand binding pockets. All of the remaining tetrahedra form a constrained Delaunay tessellation.

#### Step 4. Determination of the protein boundary for the structure

The tetrahedra with the radius of the circumscribed sphere larger than 7.5 Å can be further removed from the constrained Delaunay tessellation defined in step 3. A new boundary (blue and purple solid lines in the Figure 1), which still contains all of the C*α *atoms of the protein, is formed from the new triangles resulting from the removal of the tetrahedra and the remaining triangles on the convex hull. This boundary is called the protein boundary. The cut-off value of 7.5 Å is derived from the parameterization procedure described below and is based on the separation in size of circumscribed spheres (and hence tetrahedra) that define the surface versus the interior of the protein. The removed tetrahedra from the constrained Delaunay tessellation are candidates for the virtual atom, which is the circumscribed sphere outside the protein boundary but inside the environmental boundary. Protein space is thus partitioned into three parts defined by the protein and environmental boundaries – the C*α *atoms of the protein inside the protein boundary, the virtual atoms inside the environmental boundary but outside the protein boundary, and that occupied by the solvent outside the environmental boundary. It is noted that the protein and environmental boundaries may overlap.

#### Step 5. Computation of geometric measurements

Associated with each C*α *atom is a vector describing the distance and direction to the environmental boundary. To compute the distance and direction, the closest plane(s) to the C*α *atom, determined from the triangles on the boundary, are first selected. Then the boundary distance *P*, which will be used in the next step, is the distance from the C*α *atom to the closest plane, and the boundary direction is the normal vector of the closest plane. If there is more than one closest plane that has the same distance to the C*α *atom (for example, the distances from the C*α *atom on the environmental boundary to all its intersected planes are 0.0), the average of the normal vectors of these closest planes is taken as the atom's direction. The distance and direction of the C*α *atom to the protein boundary is computed in the same way as to the environmental boundary.

#### Step 6. Computation of geometric potential

The value of the geometric potential (GP) at each C*α *atom depends on the atom's distance to the environmental boundary and the distances and directions to neighboring C*α *atoms that are located on the protein boundary and unobstructed by other residues inside the protein boundary. This can be described as follows:

GP=P+∑neighborsPiDi+1.0×cos⁡(αi)+1.02.0     (2)
 MathType@MTEF@5@5@+=feaafiart1ev1aaatCvAUfKttLearuWrP9MDH5MBPbIqV92AaeXatLxBI9gBaebbnrfifHhDYfgasaacH8akY=wiFfYdH8Gipec8Eeeu0xXdbba9frFj0=OqFfea0dXdd9vqai=hGuQ8kuc9pgc9s8qqaq=dirpe0xb9q8qiLsFr0=vr0=vr0dc8meaabaqaciaacaGaaeqabaqabeGadaaakeaacqWGhbWrcqWGqbaucqGH9aqpcqWGqbaucqGHRaWkdaaeqbqaamaalaaabaGaemiuaafddaWgaaqaaiabdMgaPbqabaaakeaacqWGebardaWgaaWcbaGaemyAaKgabeaakiabgUcaRiabigdaXiabc6caUiabicdaWaaaaSqaaiabd6gaUjabdwgaLjabdMgaPjabdEgaNjabdIgaOjabdkgaIjabd+gaVjabdkhaYjabdohaZbqab0GaeyyeIuoakiabgEna0oaalaaabaGagi4yamMaei4Ba8Maei4CamNaeiikaGccciGae8xSdegddaWgaaqaaiabdMgaPbqabaGccqGGPaqkcqGHRaWkcqaIXaqmcqGGUaGlcqaIWaamaeaacqaIYaGmcqGGUaGlcqaIWaamaaGaaCzcaiaaxMaadaqadaqaaiabikdaYaGaayjkaiaawMcaaaaa@5EEA@

Where *P *is the distance of a given C*α *atom to the environmental boundary. The index, *i*, indicates the *i*th neighboring C*α *atom that is located on the protein boundary and unobstructed by other residues inside the protein boundary within a 10.0 Å radius. *P*_*i*_, *D*_*i*_, and *α*_*i *_are its distance to the environmental boundary, distance and relative direction to the given C*α *atom, respectively. The formula is similar to that proposed by Mancera et al. [41] to calculate the hydrophobicity of the binding site. In fact if *P *is substituted with the value for the hydrophobicity of the residue, the geometric potential is equivalent to the hydrophobicity in the binding site. Other geometric or topological measurements for *P *are possible, such as the distance to the centric of the protein [13], travel depth [1] and closeness of residues to other residues in protein residue interaction networks [32]. Finally, the value of the geometric potential for each C*α *atom is normalized to lie between 0.0 and 100.0.

#### Step 7. Construction of the virtual ligand and prediction of the ligand binding site

The tetrahedra that are labeled as candidates for virtual atoms and were discarded in step 3 are further processed to construct the final virtual ligand. First, the tetrahedra whose circumscribed sphere's center is outside the environmental boundary are removed. This procedure guarantees that all virtual atoms are within the environmental boundary. Second, the remaining tetrahedra are considered virtual atoms if their radii are larger than 7.5 Å. The cut-off value is derived from the parameterization procedure described below and is based on the separation in size of spheres (and hence tetrahedra) that define the surface versus the interior of the protein. Virtual atoms are then clustered. Two virtual atoms fall into the same cluster if their circumscribed spheres overlap. Each of the virtual atom clusters is considered a virtual ligand. The negative image of the virtual ligand is the predicted ligand binding site as identified in a similar manner to the ligand binding site for the known ligand, i.e., the C*α *atoms predicted are those where any of the atoms are within a 10.0 Å radius from any virtual atom and the line segment connecting these two atoms does not intersect with other spheres. Moreover, an overall geometric potential for each predicted binding site is calculated as the average of the geometric potentials for all C*α *atoms within the site. The average geometric potential is used to rank the predicted binding sites.

### Parameterization of the algorithm

The algorithm only requires one simple parameter – the radius of the circumscribed sphere within the protein boundary that distinguishes it from spheres within the environmental boundary but outside the protein boundary. We define a tetrahedron as solid if its four edges are formed by residues considered to be in contact. Here two amino acid residues indexed *i *and *j *are defined in contact if they have at least one pair of atoms *A*_*i *_and *A*_*j *_where the difference between *i *and *j *is one or whose distance *D*_*ij *_satisfies the following condition if |*i *- *j*| > 1:

*D*_*ij *_<= *R*_*i *_+ *R*_*j *_+ 2.0*R*_*a *_    (3)

Where *R*_*i *_and *R*_*j *_are the van der Waals radii of atom *A*_*i *_and *A*_*j*_, respectively and *R*_*a *_is the radius of the water molecule (a value of 1.4 Å is used).

**Figure 2:**
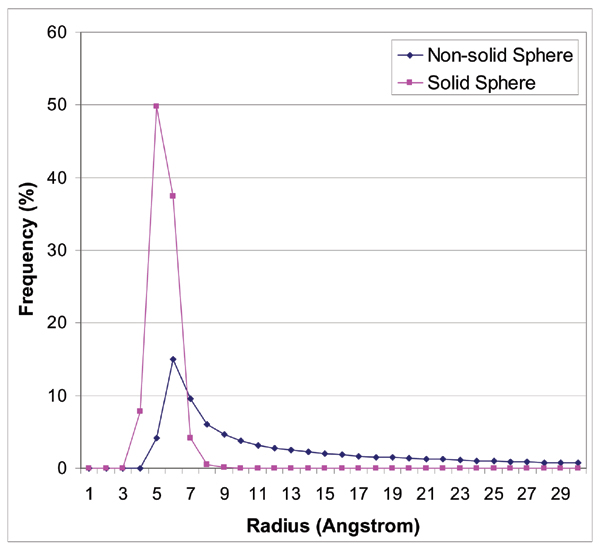
**Radius distribution of solid and non-solid circumscribed spheres from the C*α *atom Delaunay tessellation of the protein structure**. A tetrahedron is defined as solid if its four edges are formed by amino acid residues considered to be in contact (see Methods).

As shown in Figure 2, over 99.0% of solid tetrahedra have a sphere radius less than 7.5 Å. Figure 2 further shows that there is a distinct distribution between the radius of the solid and non-solid tetrahedra. They are best separated at around 7.0 Å. On the other hand, the average radius between two contacted amino acid residue as defined above in formula 3 is around 6.0 Å from the statistics analysis of PDB structures (data not shown). In order to include the water molecule in a tetrahedron formed from four residues requires a sphere of at least 7.5 Å. Thus, 7.5 Å is selected as the cut-off value for the virtual atoms. Thus, only those pockets binding to ligands larger than a water molecule are considered.

### Performance evaluation

Performance of the algorithm is evaluated by comparison to the reference binding sites on a protein by protein basis. The performance is measured by two criteria: rank accuracy and boundary sensitivity/specificity. The rank accuracy is the rank of correctly predicted sites over all predicted sites of a protein. The sensitivity and specificity are used to evaluate the residue coverage of the predicted binding site in a protein. They are defined as follows:

Sensitivity = true positives/(true positives + false negatives) × 100

Specificity = true negatives/(false positives + true negatives) × 100

**Figure 3:**
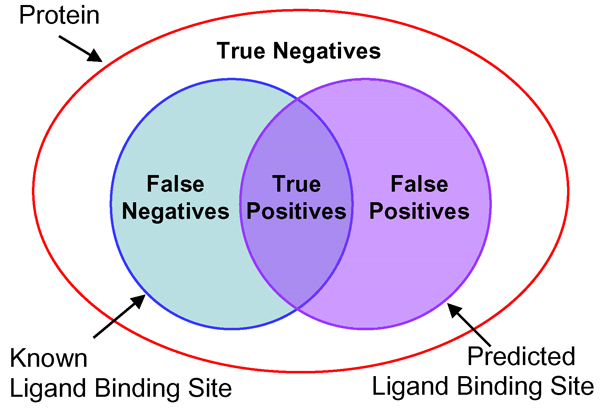
**Definition of true/false positives and true/false negatives for the predicted ligand binding site residues evaluated with respect to the referenced ligand binding site in a protein**. True and false positives are the correctly and incorrectly predicted number of binding site residues in a protein, respectively. True and false negatives are the correctly and incorrectly predicted number of non-binding site residues, respectively. They are defined for each known ligand binding site on a protein by protein basis.

**Figure 4:**
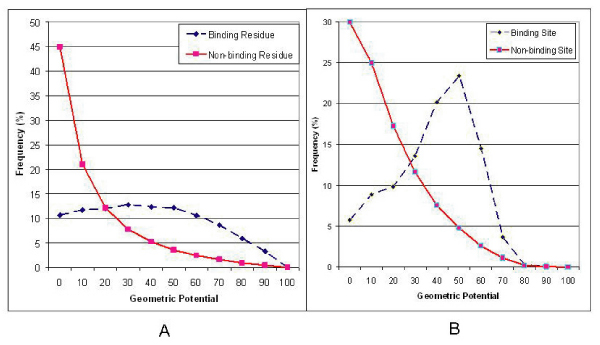
**Distribution of geometric potentials of residues that are involved and not involved in ligand binding from known protein-ligand complexes in the benchmark**. (A)single residues; (B) residue clusters that correspond to the ligand binding site and those randomly generated from the protein structure (see Methods).

**Figure 5:**
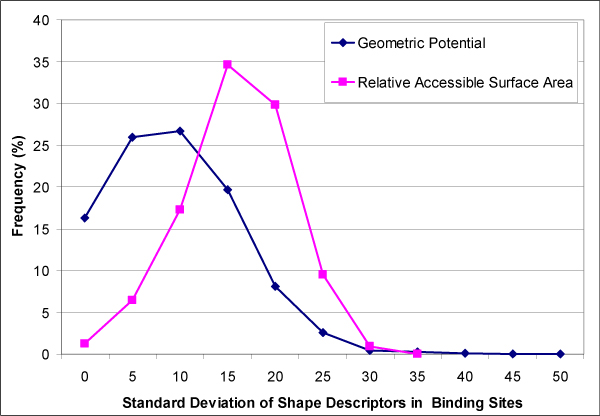
Distribution of the standard deviations of geometric potential and relative solvent accessible surface area in the binding sites, scaled between 0.0 and 100.0.

**Figure 6:**
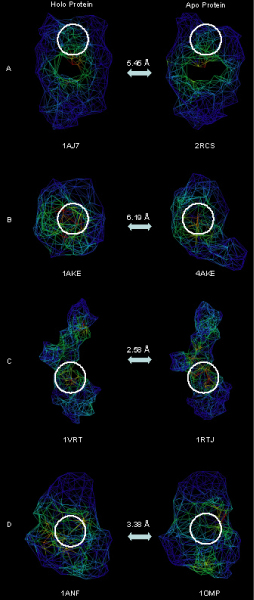
**Reduced protein structure representation with C*α *atoms and Delaunay tessellation showing the computed geometric potential**. Each vertex in the figure corresponds to a C*α *atom. The relative values of the geometric potential are color-coded, red (highest), blue (lowest). The structures shown in the left and the right columns are holo and apo proteins, respectively. The known ligand binding site is located in the white circle. The RMSD between the holo and apo protein across the whole structure is shown above the arrow. (A) Immunoglobulin 48g7 germline fab (PDB id: 1AJ7 and 2RCS); (B) Adenylate kinase (PDB id: 1AKE and 4AKE); (C) HIV-1 reverse transcriptase (PDB id: 1VRT and 1RTJ); (D) Maltodextrin binding protein (PDB id: 1ANF and 1OMP).

**Figure 7:**
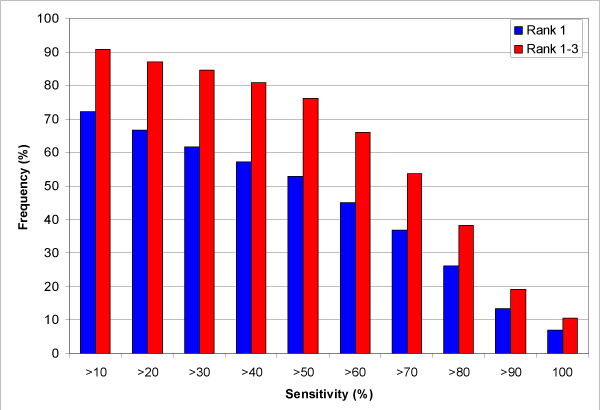
Distribution of the sensitivity for correctly predicted ligand binding sites ranked as first (blue bar) and in the top three (red bar), respectively, for all protein complexes in the benchmark.

**Figure 8:**
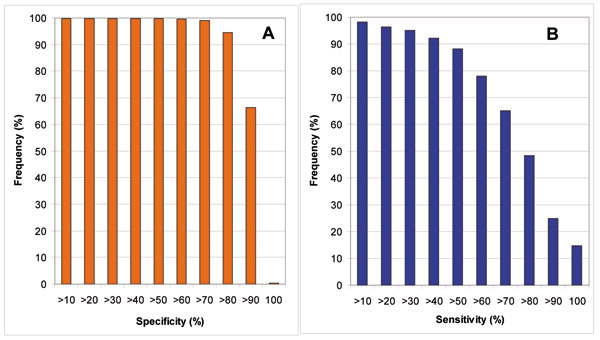
**Distribution of (A) specificity and (B) sensitivity of all predicted ligand binding sites with respect to the referenced ligand binding sites.** See Methods and Figure 3 for the definition of the specificity and the sensitivity.

**Figure 9:**
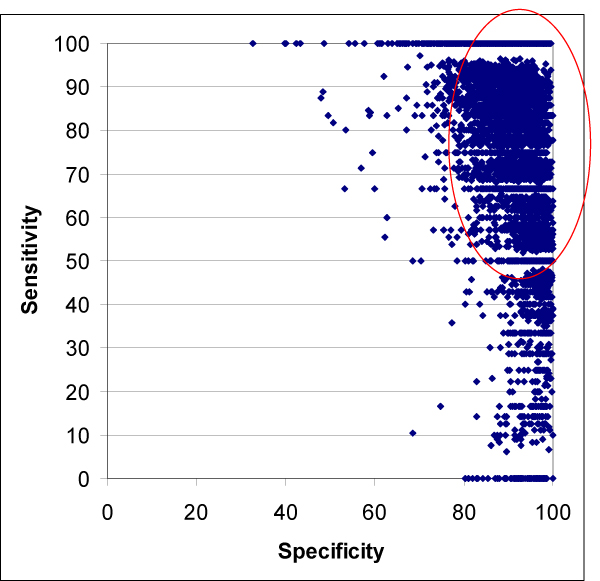
**Sensitivity vs. specificity of all predicted ligand binding sites**. Each point in the figure corresponds to a predicted ligand binding site. ~85% of predictions have sensitivity above 50% and specificity above 80%, as shown by the red circle.

**Figure 10:**
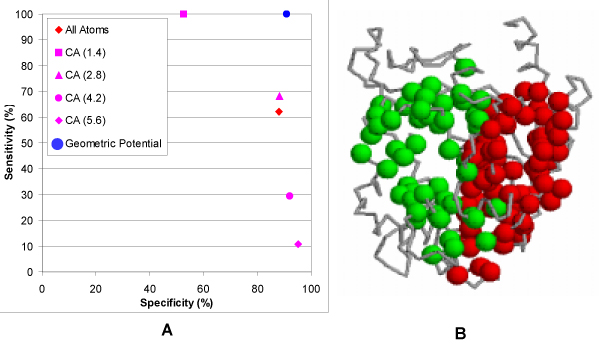
**An example of ligand binding site prediction from CASTp [29]**. (A) sensitivity vs. specificity for all atom and C*α *only representations with different probe radii by CASTp predictions [29]. As a comparison, the prediction from the geometric potential is marked as a solid blue circle. (B) The predicted two largest binding sites from CASTp [29] with C*α *only representation and probe radius 2.8 Å. The two largest pockets with similar volume and surface area are shown in the figure. The pockets colored green and red represent a known ligand binding site and a helical interface, respectively.

**Figure 11:**
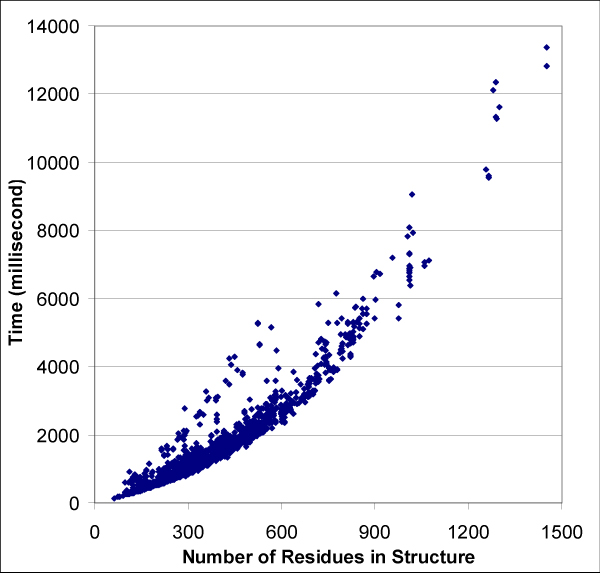
**Time complexity of the algorithm**. Times are run times on a single non-dedicated 3.0 GHz processor with 2.0 GB of RAM.

Where true and false positives are correctly and incorrectly predicted number of binding site residues in a protein, respectively. True and false negatives are correctly and incorrectly predicted number of non-binding site residues, respectively, as shown in Figure 3. Thus, the value of both the sensitivity and the specificity for a 100% accurate prediction will be 100.

## Competing interests

The authors declare that they have no competing interests.

## Authors' contributions

LX developed the concept, implemented the code, did the calculations and drafted the paper. PEB defined and directed the application of the concept and finalized the draft. Both authors read and approved the final manuscript.
